# Inanspruchnahme von Versorgungsangeboten junger Erwachsener mit ersten psychotischen Episoden im FRITZ am Urban in Berlin unter Berücksichtigung des Migrationshintergrunds

**DOI:** 10.1007/s00115-024-01777-4

**Published:** 2025-01-16

**Authors:** Miriam Bernhardt, Stefan Siebert, Johanna Baumgardt, Olga Maria Domanska, Karolina Leopold, Andreas Bechdolf

**Affiliations:** 1 Kliniken für Psychiatrie, Psychotherapie und Psychosomatik mit FRITZ am Urban & soulspace, Vivantes Klinikum Am Urban und Vivantes Klinikum im Friedrichshain, Dieffenbachstraße 1, 10967 Berlin, Deutschland; 2 Alfred Adler Gesellschaft für Individualpsychologie in Berlin e. V., Berlin, Deutschland; 3 Klinik und für Psychiatrie und Psychotherapie, Zentrum für Psychosoziale Medizin, Universitätsklinikum Hamburg-Eppendorf, Hamburg, Deutschland; 4 Wissenschaftliches Institut der AOK, Berlin, Deutschland; 5 Klinik und Poliklinik für Psychiatrie und Psychotherapie, Universitätsklinikum Carl Gustav Carus, Dresden, Deutschland; 6 Klinik für Psychiatrie und Psychotherapie, CCM, Charité Universitätsmedizin Berlin, corporate member of Freie Universität Berlin and Humboldt-Universität zu Berlin, Berlin, Deutschland; 7 Deutsches Zentrum für Psychische Gesundheit (DZPG), Berlin-Potsdam, Deutschland

**Keywords:** Unbehandelte Psychose, Versorgungswege, Frühintervention, Migration, Bildungsniveau, Untreated psychosis, Treatment pathways, Early intervention, Migration, Education level

## Abstract

**Hintergrund:**

In Deutschland liegen kaum Studien vor, die Versorgungswege im Frühverlauf von Psychosen sowie die Dauer der unbehandelten Psychosen (DUP) untersuchen und dabei den Migrationshintergrund berücksichtigen.

**Ziel der Arbeit:**

Die Studie untersucht, ob sich junge Erwachsene mit (PmM) und ohne Migrationshintergrund (PoM), die innerhalb der letzten fünf Jahre eine erste psychotische Episode erlebten oder das psychiatrische Versorgungssystem erstmalig in Anspruch genommen haben, in der Inanspruchnahme von Versorgungsangeboten und der DUP unterscheiden.

**Material und Methoden:**

Die Datenerhebung sowie Post-hoc-Analysen wurden im Rahmen einer Kohortenstudie (84 stationäre Patient*innen) am Frühinterventions- und Therapiezentrum (FRITZ) in Berlin durchgeführt.

**Ergebnisse:**

PmM mit ersten psychotischen Episoden (*n* = 38) zeigten keine signifikanten Unterschiede zu der Vergleichsgruppe (PoM, *n* = 46) in der Inanspruchnahme von Versorgungsangeboten (*p* = 0,22). Die Zeitspanne bis zum Aufsuchen eines ersten Versorgungsangebots, die Anzahl von Kontakten ins Versorgungssystem sowie die DUP wiesen zwischen PmM und PoM keine signifikanten Unterschiede auf. PmM waren überwiegend bildungsnahe junge Erwachsene, die in Deutschland aufgewachsen sind.

**Diskussion:**

Hilfsangebote im FRITZ wurden von allen Nutzenden ungeachtet ihres Migrationshintergrundes rasch aufgesucht, was für die weitere Implementierung spezialisierter Früherkennungsangebote in Deutschland spricht. Die Ergebnisse könnten auf eine Selektion der Studienpopulation zurückzuführen sein.

**Schlussfolgerung:**

Weitere Untersuchungen, die bildungsferne Personen und solche mit geringen Sprachkenntnissen einschließen, sind notwendig. Die Früherkennungsangebote und Aufklärungskampagnen sollen für diese Zielgruppe angepasst werden.

## Abstract (en)

**Background:**

In Germany, there are hardly any studies that investigated the care pathways in the early course of psychosis and the duration of untreated psychosis (DUP) and took the migration background into account.

**Objective:**

The study examined whether young adults with (PwM) and without a migration background (PoM) who had a first psychotic episode or first contact with the psychiatric care system within the last 5 years differ in their utilization of care services and DUP.

**Material and methods:**

The data collection and post hoc analyses were carried out as a part of a cohort study (84 inpatients) at the Early Intervention and Therapy Center (FRITZ) in Berlin.

**Results:**

The number of PwM (*n* = 38) who experienced a first psychotic episode did not significantly differ from the comparison group (PoM, *n* = 46) in the utilization of healthcare services (*p* = 0.22). Neither the time until seeking help from the first service nor the number of contacts with healthcare services and DUP significantly differed between PwM and Pom. The PwM were predominantly young adults with a good education background who grew up in Germany.

**Discussion:**

Users sought help at FRITZ relatively quickly, independent of their migration background, which supports further implementation of specialized early detection and intervention services in Germany. The results could be attributed to the selection of the study population.

**Conclusion:**

Further studies are needed that address people with lower education and low language skills. The early detection programs and awareness campaigns should be adapted to this target group.

## Hintergrund und Fragestellung

Junge Menschen mit psychotischen Erkrankungen nehmen häufig erst spät psychiatrische Behandlungsangebote in Anspruch, was weitreichende Konsequenzen für sie selbst, ihre Angehörigen und die Gesellschaft haben kann [19]. Ein Maß für die Zeit von der Erstmanifestation einer psychotischen Episode bis zum Beginn einer adäquaten Behandlung ist die Dauer der unbehandelten Psychose (DUP; [16]). Laut der neuesten Metaanalyse liegt die DUP international im Durchschnitt bei 42,6 Wochen, mit Werten von 38,6 Wochen in Europa und etwa 28 Wochen in Australien [22]. In Deutschland variiert die DUP je nach Studie zwischen 22 und 62 Wochen [8, 12, 13].

Die Dauer der unbehandelten Psychose wird als ein wichtiger prognostischer Faktor bei dem ersten Kontakt mit dem Hilfesystem betrachtet, wobei eine längere DUP mit geringerem Behandlungserfolg, schwererer Positiv- und Negativsymptomatik und niedrigerem Funktionalitätslevel assoziiert ist [9]. Die DUP kann von unterschiedlichen strukturellen Faktoren abhängen, die vor allem auf das Versorgungssystem zurückzuführen sind wie z. B. Aufklärungskampagnen in der Öffentlichkeit, systematisierte Zugangswege zur Versorgung oder das Vorhalten von Früherkennungsangeboten und Frühinterventionsprogrammen [13, 15, 23]. Darüber hinaus kann die DUP durch Patient*innenmerkmale, krankheitsbezogene sowie durch Merkmale des sozialen Umfelds und soziodemografische Faktoren beeinflusst werden [24]. So können ethnische Herkunft (z. B. „non-white“, „black-caribbaen“) oder ein Migrationshintergrund mit einer längeren DUP assoziiert sein [5, 20, 22, 25]. Personen mit Migrationshintergrund (PmM) suchten in Vorstudien später professionelle Hilfe [16, 25] und nahmen das Gesundheitssystem weniger in Anspruch als Personen ohne Migrationshintergrund (PoM; [5, 16]).

In Deutschland liegen bis dato zwei Studien vor, die Versorgungswege im Frühverlauf von Psychosen unter Berücksichtigung des Migrationshintergrunds untersuchten [26, 31]. In einer frühen Studie am Kölner Früherkennungszentrum wurde für PmM und mit einem erhöhten Psychoserisiko eine geringere Inanspruchnahme eines Früherkennungsangebotes beobachtet [26]. Eine weitere Studie am Früherkennungs- und Therapiezentrum (FRITZ) in Berlin zeigte, dass vor allem Migrant*innen erster Generation seltener eine stationäre (Vor‑)Behandlung in Anspruch nahmen [31]. In beiden Studien wurden jedoch keine Daten zur DUP für PmM berichtet.

Vor diesem Hintergrund untersucht die vorliegende Studie, ob Unterschiede in der Inanspruchnahme von Versorgungsangeboten und der DUP bei jungen Erwachsenen mit ersten psychotischen Episoden, die eine spezialisierte vollstationäre Behandlung im FRITZ in Berlin wahrnahmen, hinsichtlich des Migrationshintergrunds bestehen. Spezifiziert wurden folgende Hypothesen: PmM im Vergleich zu PoM …

- weisen vom Auftreten unspezifischer Symptome eine längere Zeitspanne bis zum Aufsuchen eines ersten Hilfsangebotes auf,
- haben mehr Kontakte ins Hilfesystem bis zur adäquaten Behandlung,
- weisen eine längere DUP auf.

## Studiendesign und Untersuchungsmethoden

### Datengrundlage

Die Daten der vorliegenden Untersuchung stammen aus einer 1‑Jahres-Kohortenstudie, die im FRITZ erhoben wurde [27]. Es handelt es sich somit um Post-hoc-Analysen. Das FRITZ wurde speziell für junge Menschen mit ersten psychotischen Krisen am Vivantes Klinikum Am Urban entwickelt und folgt nationalen und internationalen Behandlungsleitlinien [1, 7, 10]. Das Behandlungsprogramm beinhaltet psychotherapeutische und soziotherapeutische Interventionen, „individual placement and support“, Genesungsbegleitung sowie psychopharmakologische Behandlung [3, 27]. Auf das Angebot wird durch eine jugendfreundliche Homepage (https://fritz-berlin.de/), allgemeine Öffentlichkeits- und Awarenessarbeit in Schulen und Jobcentern sowie durch eine enge Vernetzung mit den psychosozialen Angeboten im Bezirk hingewiesen.

Die Studienteilnehmenden der Kohortenstudie wurden von Dezember 2015 bis Mai 2018 im vollstationären Setting rekrutiert (für weitere Details Siebert et al. [27]). Von den ursprünglichen 95 Studienteilnehmenden der Studie konnten 84 Personen mit Angaben zum Migrationshintergrund in die Analysen eingeschlossen werden. Entsprechend der Definition früher psychotischer Erkrankungen galten folgende Einschlusskriterien:

1. Vorliegen einer psychotischen Erkrankung (International Statistical Classification of Diseases and Related Health Problems 10, ICD-10): substanzinduzierte psychotische Störungen (F1x.5), Schizophrenie, schizotype und wahnhafte Störungen (F2x.x), manische Episoden mit psychotischen Symptomen (F30.2), bipolare affektive Störung, manische Episode oder schwere depressive Episode mit psychotischen Symptomen (F31.2/F31.5) und schwere depressive Episoden mit psychotischen Symptomen (F32.3/F33.3);
2. der Beginn der psychotischen Episode oder die initiale Vorstellung im Gesundheitssystems aufgrund psychischer Probleme lag nicht mehr als 5 Jahre zurück, was in Übereinstimmung mit Bird et al. definiert wurde [4];
3. die Teilnahme am vollstationären FRITZ-Therapieprogramm;
4. ausreichende intellektuelle und deutschsprachige Fähigkeiten sowie die schriftliche Einwilligung nach Aufklärung. Als adäquate Behandlung einer ersten psychotischen Episode wurde eine medikamentöse antipsychotische Therapie und spezifische psychotherapeutische Interventionen definiert [7, 14].

Das Ethikvotum für die Studie wurde am 24.02.2015 durch die Ethikkommission der Charité Campus Benjamin Franklin erteilt (Antragsnummer EA4/026/15). Die Studie wurde beim Deutschen Register Klinischer Studien registriert (DRKS-ID: DRKS00024351).

### Erhebungsinstrumente

Alle Studienoutcomes wurden zu Behandlungsbeginn durch geschulte Bezugstherapeut*innen (Ärzt*innen und Psycholog*innen) und wissenschaftliche Mitarbeiter*innen erhoben.

Die Diagnose wurde unter Zuhilfenahme eines halbstrukturierten Interviews (ICD-10-Symptom-Rating) nach ICD-10 gestellt [11]. Die Inanspruchnahme von Versorgungsangeboten seit dem Auftreten erster Symptome wurde retrospektiv durch eine an das deutsche Gesundheitssystem angepasste Version des „pathway to care“ erfragt [29].

**Abb. 1:**
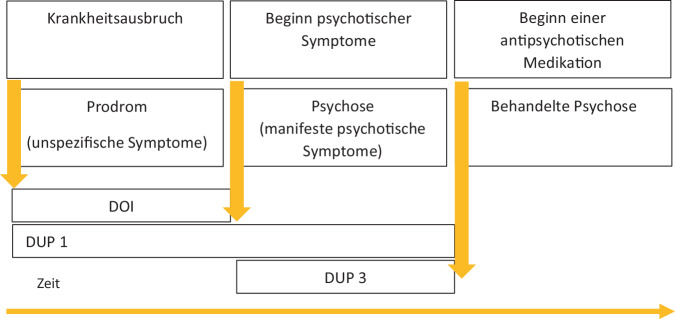
Differenzierte Darstellung für die Dauer der unbehandelten Psychose. *DOI* Krankheitsdauer, *DUP* Dauer der unbehandelten Psychose

Die Dauer der unbehandelten Psychose (DUP) wurde mittels der deutschen und angepassten Version des Nottingham Onset Schedule (NOS) erfasst [28]. Definiert und identifiziert wurden folgende Zeitspannen (Abb. 1):

- die Krankheitsdauer (DOI) als Zeitspanne von Beginn des Prodroms (unspezifische Symptome) bis zum Beginn der manifesten Psychose (Diagnosekriterien erfüllt);
- die Dauer der unbehandelten Psychose (DUP1, inkl. Prodrom) als Zeitspanne von Beginn des Prodroms bis zum Beginn einer antipsychotischen Medikation in adäquater Dosis und regelmäßiger Einnahme;
- die Dauer der unbehandelten Psychose (DUP3, manifeste Psychose) als Zeitspanne zwischen dem Erleben erster psychotischer Symptome bis zum Beginn einer antipsychotischen Medikation in adäquater Dosis und regelmäßiger Einnahme.

Der Migrationshintergrund wurde über den Geburtsort, das Alter zum Zeitpunkt der Migration, die Muttersprache sowie die Geburtsorte der Eltern erfasst. Entsprechend der Definition des Statistischen Bundesamt ist eine „Person mit Migrationshintergrund“ ein Mensch, der im Ausland geboren wurde und nach Deutschland zugezogen ist (Migrationshintergrund 1. Generation, MeG) oder in Deutschland geboren wurde und zumindest ein nach Deutschland eingewandertes Elternteil hat (Migrationshintergrund 2. Generation, MzG; [6]). Um explorativ den Einfluss der Sprache auf die DUP zu prüfen, wurden PmM in drei Gruppen eingeteilt:

- Deutsch als erste Muttersprache,
- andere Muttersprache,
- Deutsch und eine weitere Muttersprache (bilingual).

### Statistische Analysen

Es handelt sich um Post-hoc-Analysen. Die Hypothesen wurden mittels nichtparametrischer Verfahren (Mann-Whitney-U-Tests, χ^2^-Tests [zweiseitig], exakte Tests nach Fischer, Kruskal-Wallis-Test) mit einem Signifikanzlevel von *p* < 0,05 geprüft, wenn das Kriterium der Normalverteilung nicht zutraf bzw. Zielvariablen kategorial vorlagen. Für den Vergleich der DUP zwischen mehr als zwei Gruppen wurden Varianzanalysen berechnet.

## Ergebnisse

Von den 84 Studienteilnehmenden gaben 45,3 % (*n* = 38) an, einen Migrationshintergrund zu haben. Davon waren knapp ein Drittel (*n* = 12) Personen mit MeG und über zwei Drittel (*n* = 26) mit MzG. Das durchschnittliche Alter der Studienteilnehmenden lag bei 26,2 ± 5,5 Jahren; 40,5 % (*n* = 34) waren weiblich. Der überwiegende Teil der Teilnehmenden hatte (Fach‑)Abitur und befand sich in Ausbildung/Studium (Tab. 1).

**Tab. 1:** Beschreibung der Studienteilnehmenden (*n* = 84)

	PoM (*n* = 46)	PmM (*n* = 38)
	*n* (%)	*n* (%)
*Geschlecht*		
Weiblich	17 (37,0)	17 (44,7)
*Familienstand*		
Ledig	40 (88,9)	30 (78,9)
In Partnerschaft	5 (11,1)	8 (21,1)
*Schulabschluss*		
Schüler*in	2 (4,4)	0
(Erweiterter) Hauptschulabschluss	5 (11,1)	8 (21,1)
Mittlere Reife	14 (31,1)	8 (21,1)
(Fach‑)Abitur	23 (51,1)	22 (57,9)
Kein Abschluss	1 (2,2)	0
*Bildungsjahre*	*MW (SD)*	*MW (SD)*
	13,8 (3,3)	14,9 (3,7)
*Diagnosen*		
Drogeninduzierte psychotische Störung	21 (45,7)	17 (44,7)
Affektive Störung mit psychotischen Symptomen	8 (17,4)	1 (2,6)
Schizophrenie, schizotype und wahnhafte Störungen	17 (36,9)	20 (52,7)

*MW* Mittelwert, *SD* Standardabweichung, *PmM* Personen mit Migrationshintergrund, *PoM* Personen ohne Migrationshintergrund

Daten zu Familienstand/Schulabschluss fehlen bei 1 PoM (2,2 %)

**Abb. 2:**
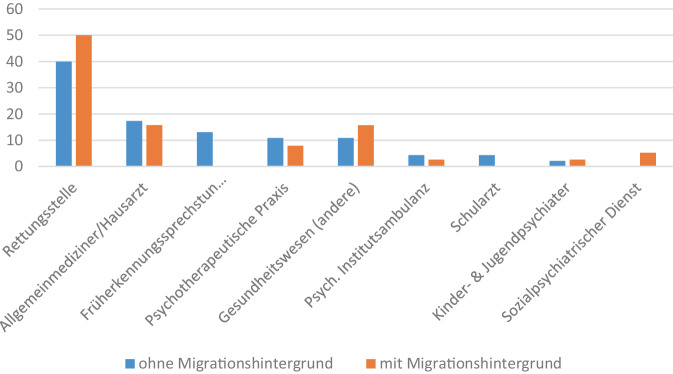
Inanspruchnahme von Versorgungsangeboten im ersten Hilfesuchversuch (in %; *n* = 84)

Die Inanspruchnahme der Versorgungsangebote seit dem Auftreten von Prodromalsymptomen von Personen mit und ohne Migrationshintergrund ist in Abb. 2 dargestellt. PmM suchten häufiger die Rettungsstelle oder andere Anlaufstellen des Gesundheitswesens beim ersten Hilfeversuch auf. Signifikante Unterschiede in Inanspruchnahme dieser und der weiteren Versorgungsangebote zwischen Personen mit und ohne Migrationshintergrund wurden nicht beobachtet (χ^2^(8) = 10,66, *p* = 0,22).

Ebenso zeigten sich keine statistisch signifikanten Unterschiede zwischen Personen mit und ohne Migrationshintergrund in der Zeitspanne vom Auftreten erster (unspezifischer) Symptome bis zum Aufsuchen eines ersten Hilfsangebots (Median: 23 vs. 30 Tage) und in der Anzahl von Kontakten zu Versorgungsangeboten (ca. 3) bis zur adäquaten Behandlung (Tab. 2). Dennoch ist anzumerken, dass die durchschnittliche Anzahl der Tage bis zu der ersten professionellen Hilfe bei PmM deutlich höher ist.

**Tab. 2:** Inanspruchnahme von Versorgungsangeboten und DUP in Abhängigkeit vom Migrationshintergrund (*n* = 84)

	PoM (*n* = 46)		PmM (*n* = 38)		Teststatistik, *p*-Werte
**–**	**MD (R)**	***n***	**MD (R)**	***n***	**–**
*DUP in Tagen*					
DOI	213 (2–4748)	39	258 (1–2192)	34	U = 655,00; z = −0,09; *p* = 0,93
DUP1	289 (2–4748)	40	380 (7–3650)	36	U = 686,00; z = −0,35; *p* = 0,72
DUP3	28 (0–396)	37	28 (0–1461)	33	U = 562,00; z = −0,57; *p* = 0,57
**–**	**MW (SD)**	***n***	**MW (SD)**	***n***	**–**
Anzahl von Kontakten zu Versorgungsangeboten einschließlich der Behandlung im FRITZ	2,76 (1,02)	46	2,50 (1,18)	38	F (1,84) = 1,19; *p* = 0,28
Zeitspanne vom Auftreten erster (unspezifischer) Symptome bis zum Aufsuchen des ersten Hilfsangebots in Tagen	133,39 (262,53)	44	278,14 (724,42)	37	U = 780,00; z = −032; *p* = 0,75

*DUP* Dauer der unbehandelten Psychose; *DOI* Krankheitsdauer, als Zeitspanne von Beginn des Prodroms (unspezifische Symptome) bis zum Beginn der manifesten Psychose (Diagnosekriterien erfüllt); *DUP1* Dauer der unbehandelten Psychose als Zeitspanne von Beginn des Prodroms bis zum Beginn einer antipsychotischen Medikation; *DUP3* Dauer der unbehandelten Psychose als Zeitspanne zwischen dem Erleben erster psychotischer Symptome (manifeste Psychose) bis zum Beginn einer antipsychotischen Medikation; *MW* Mittelwert, *MD* Median, *SD* Standardabweichung, *R* Range

### Dauer der unbehandelten Psychose

Personen mit und ohne Migrationshintergrund unterscheiden sich nicht signifikant in den unterschiedlichen Zeitspannen der DUP, also DOI, DUP1 und DUP3 (Tab. 2). Die Differenzierung zwischen Personen ohne Migrationshintergrund, Personen mit MeG und MzG zeigt auch keine Unterschiede für die untersuchten Zeitspannen in der DUP (Tab. 3).

**Tab. 3:** Dauer der unbehandelten Psychose in Abhängigkeiten vom Migrationshintergrund und Differenzierung nach Muttersprache(n) für Personen mit Migrationshintergrund

Migrationsstatus	PoM (*n* = 46)		MeG (*n* = 12)		MzG (*n* = 26)		Teststatistik, *p*-Werte
	MW(SD)	*n*	MW(SD)	*n*	MW(SD)	*n*	
*DUP in Tagen*							
DOI	715 (1145,98)	39	500,55 (5576,86)	11	463,96 (612,71)	23	F (2,73) = 0,60; *p* = 0,55
DUP1	816,33 (1185,50)	40	584,33 (581,25)	12	721,21 (915,99)	24	F (2,76) = 0,25; *p* = 0,78
DUP3	77,84 (107,94)	37	133,75 (205,43)	12	106,74 (211,95)	21	F (2,70) = 0,73; *p* = 0,49
*PmM: Muttersprache*	Deutsch (*n* = 13)	**–**	Andere (*n* = 15)	**–**	Bilingual (*n* = 10)	**–**	**–**
*DUP in Tagen*							
DOI	455,73 (621,61)	11	499,08 (579,66)	13	467,60 (640,18)	10	F (2,34) = 0,02; *p* = 0,98
DUP1	494,73 (708,733)	11	771,53 (977,01)	15	675,58 (813,53)	10	F (2,36) = 0,39; *p* = 0,68
DUP3	82,22 (121,77)	9	111,93 (196,15)	14	228,50 (456,62)	10	F (2,33) = 0,72; *p* = 0,50

*MeG* Personen mit Migrationshintergrund der ersten Generation; *MzG* Personen mit Migrationshintergrund der zweiten Generation; *DUP* Dauer unbehandelter Psychose; *DOI* Krankheitsdauer, als Zeitspanne von Beginn des Prodroms (unspezifische Symptome) bis zum Beginn der manifesten Psychose (Diagnosekriterien erfüllt); *DUP1* Dauer der unbehandelten Psychose als Zeitspanne von Beginn des Prodroms bis zum Beginn einer antipsychotischen Medikation; *DUP3* Dauer der unbehandelten Psychose als Zeitspanne zwischen dem Erleben erster psychotischer Symptome (manifeste Psychose) bis zum Beginn einer antipsychotischen Medikation

Explorativ wurde auch der Einfluss der Muttersprache in der Personengruppe mit Migrationshintergrund auf die DUP untersucht. Personen mit Migrationshintergrund und der Muttersprache Deutsch, einer anderen Muttersprache oder bilingualer Muttersprache zeigen keine Unterschiede in den untersuchten Zeitspannen der DUP.

## Diskussion

Anders als in vorangegangenen Studien [5, 16, 24] zeigten sich in der vorliegenden Studie keine signifikanten Unterschiede zwischen Personen mit und ohne Migrationshintergrund in der Inanspruchnahme erster Versorgungsangebote. Diese Ergebnisse könnten auf eine besonders effektive Öffentlichkeitsarbeit und Vernetzung des FRITZ mit den psychosozialen Angeboten im Bezirk zurückgeführt werden, die auch PmM gut erreichen. Zuerst die Rettungsstelle aufzusuchen, entspricht einem Inanspruchnahmeverhalten, das bereits früher in Berliner Bezirken mit hohem Migrationsanteil für somatische Fachgebiete spezifiziert wurde [18]. Die beobachtete ausschließliche Inanspruchnahme von Früherkennungssprechstunden von PoM entspricht dem Vorbefunden aus Nordrhein-Westfalen [26].

Divergent zu vorangegangen Studien unterschieden sich PmM und PoM nicht hinsichtlich der Zeitspanne vom Auftreten erster unspezifischer Symptome bis zum Aufsuchen eines ersten Hilfsangebotes, der Anzahl von Kontakten ins Hilfesystem [5, 16, 25] sowie der DUP [5, 25], sodass keine der gestellten Hypothesen im Rahmen der Post-hoc-Analyse bestätigt werden kann. Fehlende Unterschiede zwischen Personen mit und ohne Migrationshintergrund könnten auch auf ein ähnliches (hohes) Bildungs- und Sprachniveau (Einschlusskriterium) in beiden Gruppen zurückgeführt werden.

Auch der hohe Anteil des MzG könnte mit einer geringeren DUP assoziiert sein, da in einer Vergleichsstudie ein MeG mit einer längeren DUP einhergeht [21]. Im Vergleich zu gleichaltrigen PmM im Versorgungssektor innerhalb des Rekrutierungszeitraums (55 %; [2]) ist der Anteil der PmM (45,3 %) in der vorliegenden Studie unterrepräsentiert. All diese Aspekte könnten einen Selektionsbias einer bildungsnahen und sprachkompetenten Studienpopulation zugunsten einer zügigen Inanspruchnahme der Versorgung darstellen. Teststärkeprobleme aufgrund des geringen Stichprobenumfangs können nicht ausgeschlossen werden. Unsere Ergebnisse sind daher mit Vorsicht zu verallgemeinern.

Beachtenswert scheint die geringe Zeitspanne bis zum Aufsuchen eines ersten Hilfsangebots von ca. 29 Wochen in der Studienpopulation im Vergleich zu 48 Wochen in einer anderen deutschen Studie [12]. Zudem ist die Zeitspanne zwischen dem Erleben der ersten psychotischen Symptome bis zum Beginn einer antipsychotischen Medikation mit einem Median von 28 Tagen deutlich kürzer als die nationalen Ergebnisse von ca. 22 Wochen [13] und internationalen Ergebnissen von 14 Wochen [22], die vermutlich durch ein akuteres Krankheitsgeschehen in städtischen Regionen erklärt werden könnte [24, 30]. Ein akuteres Krankheitsgeschehen könnte innerhalb dieser Stichprobe durch den hohen Anteil an substanzinduzierten psychotischen Störungen (45,2 %) gestützt werden. Nicht zuletzt könnte die Vielfalt psychosozialer Angebote im Bezirk als möglicher Einflussfaktor für eine zügige Inanspruchnahme betrachtet werden.

### Limitationen

Bei der Studienpopulation handelt sich um eine selektierte Stichprobe, die kaum Personen mit geringem Bildungsniveau oder geringen Sprachkenntnissen aufweist (Ausschlusskriterium). In internationalen Studien wird betont, dass diese Faktoren mögliche Unterschiede zwischen Personen mit und ohne Migrationshintergrund beeinflussen und dies in zukünftigen Untersuchungen berücksichtigt werden sollte [17]. Die Darstellung der Ergebnisse der initialen Anlaufstellen hätte differenziert nach Krankheitsstadium (unspezifische Symptomatik vs. manifeste psychotische Symptome) erfolgen können, da das Aufsuchen eines Versorgungsangebotes wahrscheinlich je nach Zeitpunkt im Krankheitsverlauf variiert, was mit einem Migrationshintergrund in Wechselwirkung stehen könnte. Auch eine Differenzierung hinsichtlich von Diagnosegruppen z. B. Schizophrenie vs. substanzinduzierte psychotische Störungen könnte mögliche Unterschiede im Hilfesuchverhalten und der Inanspruchnahme aufdecken.

## Fazit für die Praxis

- Hilfsangebote wie das FRITZ (Früherkennungs- und Therapiezentrum) werden im Vergleich mit anderen Studien rasch (kurze Dauer der unbehandelten Psychosen) und gleichermaßen von Personen mit und ohne Migrationshintergrund angenommen, was dafür spricht, spezialisierte Angebote stärker in der Routineversorgung zu etablieren.
- Die geringe Anzahl von Menschen mit niedrigem Bildungsniveau und geringen Sprachkenntnissen in der Studie weist auf die Notwendigkeit hin, diese Gruppe bei zukünftigen spezialisierten Früherkennungs- und Behandlungsangeboten sowie in Aufklärungskampagnen besonders zu berücksichtigen.

## Data Availability

Die Studie wurde beim Deutschen Register Klinischer Studien registriert (DRKS-ID: DRKS00024351).
